# Ischemic Stroke After Cochlear Implantation in a Patient With an Undiagnosed Mitochondrial Disorder

**DOI:** 10.7759/cureus.83485

**Published:** 2025-05-04

**Authors:** Peter Kullar, Ki Wan Park, Adam Kaufman, Peter L Santa Maria

**Affiliations:** 1 Department of Otolaryngology-Head and Neck Surgery, Stanford Hospital, Palo Alto, USA

**Keywords:** cochlear implant (ci) surgery, genetic risk factors, hearing loss, neuro-otology, otolaryngology

## Abstract

Limited reports exist on cochlear implantation in patients with syndromic hearing loss due to mitochondrial disorders. Among mitochondrial disorders, mitochondrial encephalopathy, lactic acidosis, and stroke-like episodes (MELAS) is a notable example. MELAS is a disorder characterized by myopathy, seizures, diabetes, and focal neurological deficits, including sensorineural hearing loss. Careful optimization of risk factors and perioperative anesthetic considerations must be taken to avoid complications unique to MELAS, including metabolic and cardiac instability and an increased risk of stroke. Herein, we present the case of a 44-year-old woman with a history of bilateral sensorineural hearing loss who underwent a routine second side cochlear implantation which was complicated by ischemic stroke in the setting of severe intracranial atherosclerosis. Following the ischemic stroke, the patient underwent genetic testing, which confirmed a diagnosis of MELAS.

## Introduction

Mitochondrial encephalopathy, lactic acidosis, and stroke-like episodes (MELAS) is a rare mitochondrial disorder that can lead to various systemic manifestations, including stroke-like episodes, epilepsy, diabetes, short stature, myopathy, and hearing loss. Stroke-like episodes result in increased T2-weighted signal areas on brain magnetic resonance imaging (MRI) that do not correspond to a typical vascular distribution and hence are termed "stroke-like". Histological analysis of muscle biopsies reveals ragged red fibers, and biochemically, the condition is typified by lactic acidemia [1].

The most common variant associated with MELAS is the mitochondrial DNA (mtDNA) variant m.3243A>G in the MT-TL1 gene, which is reported in about 80% of patients with MELAS. Other pathogenic variants resulting in MELAS include m.3271T>C and m.3252A>G in MT-TL1 and m.13513G>A in MT-ND5 [1]. Hearing impairment is common among patients with mitochondrial disorders, including MELAS, affecting up to 58% of individuals and varying in severity from mild to severe [2]. Profound hearing loss, which can present as a solitary symptom or in the setting of an underlying syndrome, warrants evaluation for cochlear implant candidacy. However, cochlear implantation in patients with mitochondrial disorders poses unique challenges due to potential perioperative complications. Cochlear implantation in the setting of MELAS has been rarely reported in the literature, with limited outcomes on surgical complications [3-5]. We discuss the unique challenges of treating a patient with undiagnosed MELAS and the special considerations regarding diagnosis, surgical risks, and perioperative management.

## Case presentation

A 44-year-old woman with a history of bilateral sensorineural hearing loss (SNHL), type 2 diabetes mellitus (T2DM) requiring insulin, spontaneous abortion, hyperlipidemia, hypertension, short stature, and alopecia totalis presented to our tertiary care neurotology clinic for a hearing loss evaluation. There was no prior genetic testing or family genetic history available. The patient gradually developed profound bilateral SNHL, which began shortly after childbirth and progressed over the past two decades. She had undergone an uncomplicated right cochlear implant one year prior at our institution. She was a high performer with her first cochlear implant (AzBio and consonant-nucleus-consonant (CNC) scores 92% and 80% at the six-month follow-up, respectively). Given her satisfaction with her initial implant, she was interested in a contralateral cochlear implant to improve her speech in noise and localization abilities.

On the day of surgery, the patient was surprisingly hyperglycemic after an overnight fast to 282 mg/dl (reference range: 70-140 mg/dl), which required insulin administration. Once euglycemia was achieved and no signs of acute metabolic decompensation were present, our anesthesia team deemed surgery safe to proceed. Anesthesia was maintained with propofol and remifentanil. After induction, there was a 10-minute episode of hypotension with mean arterial pressure as low as 48 mmHg requiring 550 mcg of phenylephrine and 6 units of vasopressin for resolution. This episode is notable given that autonomic dysfunction and hemodynamic instability are known complications in MELAS. This pattern, also observed during her prior surgery, may retrospectively have indicated an underlying mitochondrial disorder. There were no surgical intraoperative concerns, and the cochlear implant was successfully inserted.

**Figure 1:**
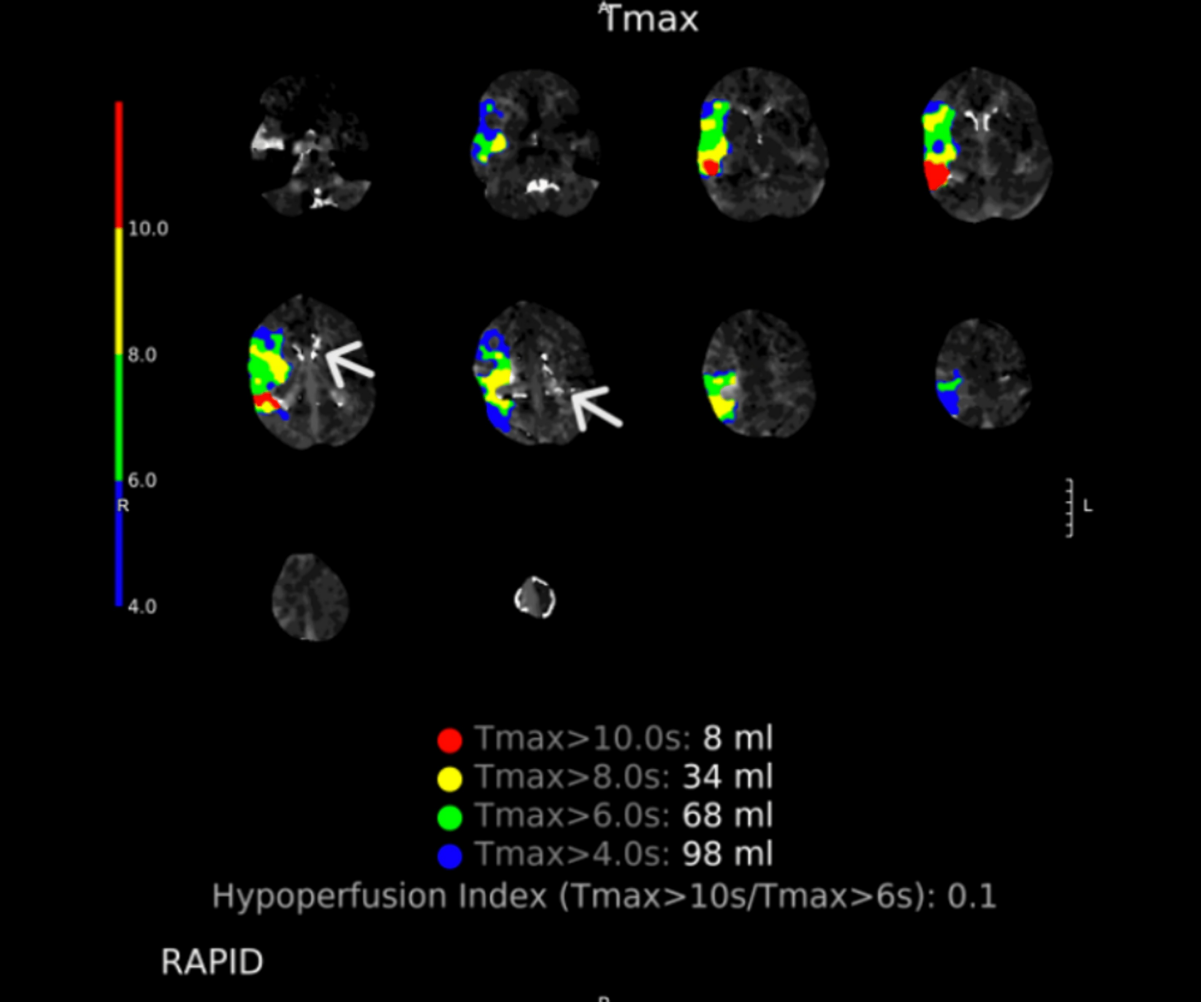
Computed tomography angiogram demonstrating perfusion defect at the right M1 segment of the right middle cerebral artery. White arrows represent calcifications in the thalamus and basal ganglia.

In the post-anesthesia care unit, the patient had a delayed emergence and was less interactive than is typical for most patients. However, this was also noted after her prior cochlear implantation surgery. Four hours after the surgery was completed, she was fully alert, and it was noticed that she had left arm weakness, dysarthria, and right gaze preference. A stroke code was called, and a computed tomography angiogram (CTA) of the head and neck was performed. It revealed a complete occlusion of the right middle cerebral artery (MCA) at the M1 segment (Figure 1). Intriguingly, in comparison with her initial preoperative computed tomography (CT) scan, she had developed significant cerebral calcifications within the thalamus and the basal ganglia. These calcifications, which were not noted on prior imaging, are consistent with Fahr's disease, a condition that can be associated with mitochondrial dysfunction.

However, given that more than six hours had elapsed since symptom onset (including the surgical time and time to regain consciousness), the patient was outside the window for tissue plasminogen activator (tPA) administration.

The penumbra seen on CTA indicated she could potentially benefit from a thrombectomy with neurointerventional radiology. The patient was re-anesthetized under propofol, and angiography showed diffuse severe intracranial atherosclerotic disease. After the thrombus was removed, the MCA re-stenosed repeatedly, requiring an intra-arterial integrilin bolus and integrilin infusion. The patient was placed on an aggressive anticoagulation protocol to help reduce the risk of re-stenosis. The recurrent re-stenosis was suggestive of an underlying vascular pathology, possibly linked to MELAS-related mitochondrial angiopathy.

Her intensive care unit (ICU) course was complicated by significant and prolonged acidosis requiring dialysis. Additionally, she was found to have organic acids in her urine. Given this unusual presentation, mitochondrial pathology was suspected, prompting genetic consultation. Consequently, metabolically toxic agents, such as propofol, which is known to increase the risk of propofol infusion syndrome in mitochondrial disorders, were avoided. 

Mitochondrial sequencing revealed that the patient had the m.3243A>G (tRNA Leu (UUR)) variant with 24.7% heteroplasmy (proportion of mutated mitochondrial DNA as a proportion of total mitochondrial DNA), consistent with MELAS. The detected heteroplasmy level is within the range associated with systemic manifestations of MELAS, including cerebrovascular events [1]. After the patient was stabilized, she was discharged to an acute rehab facility. A full genetic workup of maternal family members is currently being pursued, given the matrilineal inheritance of mitochondrial disorders.

## Discussion

MELAS is one of the most commonly inherited mitochondrial disorders with multiorgan involvement. There are limited case reports of cochlear implantation in patients with mitochondrial disorders. While existing literature describes management in the setting of known mitochondrial pathology, to our knowledge, this is one of the first reported cases where MELAS was diagnosed due to postoperative complications [3,4,6,7]. We discuss the special considerations regarding diagnosis and management required for patients with MELAS. 

SNHL is a frequent manifestation of MELAS, with reported prevalence ranging from 30% to 75% [1]. Mitochondrial disorders exhibit a threshold effect, whereby mutation load correlates with symptom severity. However, the correlation is non-linear due to differences in tissue-specific heteroplasmy levels and other modifying genetic or environmental factors. Limited studies suggest that the atrophy of the stria vascularis in the cochlea is primarily responsible for hearing loss in MELAS, while there is good preservation of the organ of Corti and the spiral ganglion [8-10]. The degree of hearing loss is linearly associated with the mutation load found in the skeletal muscle, which acts as a surrogate for mutation burden elsewhere in the body [10].

Other phenotypic manifestations of MELAS include recurrent headaches, tonic-clonic seizures, muscle weakness/exercise intolerance, stroke-like episodes, diabetes, and persistent lactic acidemia first appearing between the ages of two and 40 years [1].

The diagnosis is typically based on clinical symptoms, abnormal muscle biopsy, and confirmed genetic testing, following established diagnostic criteria [11]. Diagnosis, however, can be challenging, given the wide genotype-phenotype variation. Our patient did not meet the criteria for diagnosis prior to the identification of the pathological variant. Preoperatively, her myriad of symptoms seemed unrelated as she did not report any family history. During her extensive workup in the ICU, collateral information was gathered from other family members that her mother had a history of early-onset strokes and dementia, her brother had diabetes, and her teenage son had very short stature. Given MELAS's maternal inheritance pattern, the history of early-onset strokes and dementia in the patient's mother is particularly relevant.

Cochlear implants are a therapeutic option for patients with MELAS presenting with profound SNHL. There have only been nine reported cases of cochlear implantation in patients with known MELAS in the literature [3-7]. These case reports suggest excellent outcomes with improved speech perception and hearing abilities, but data on surgical complications in this unique patient population are very limited. Similarly, long-term data on implant stability and performance in MELAS remain scarce. Consistent with prior studies, our patient was a high performer who was satisfied with outcomes following her initial cochlear implant on the right side.

**Figure 2:**
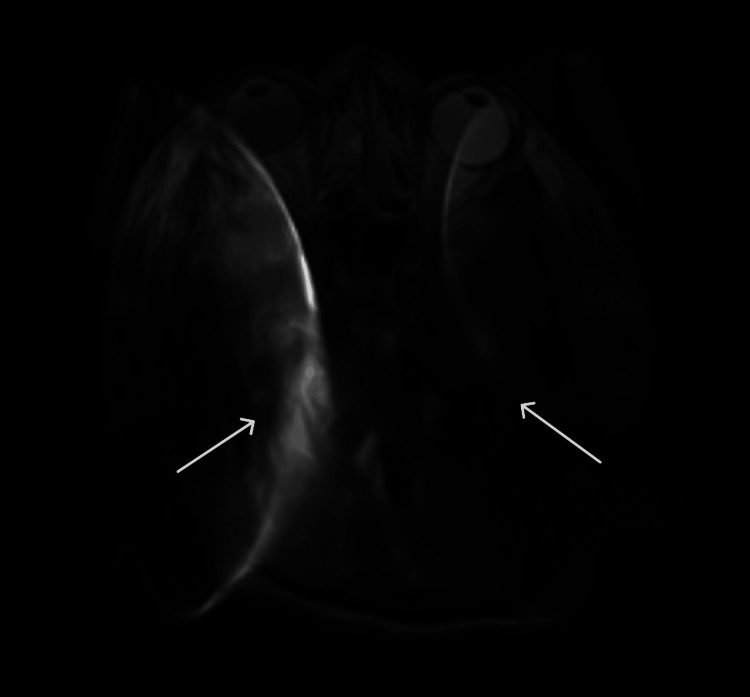
Axial T2-FLAIR MRI of the brain without contrast demonstrating signal artifact from bilateral cochlear implants. Arrows point to significant artifact resulting from the magnetic interaction of the bilateral cochlear implants. T2-FLAIR: T2-weighted fluid-attenuated inversion recovery; MRI: magnetic resonance imaging

Her bilateral cochlear implants further complicated her ICU stay and the ability to neuro-prognosticate. Although both implants were MRI compatible, there was significant artifact over large portions of the brain (Figure 2). A single implant can cause shadowing, limiting visibility, but there appeared to be a magnetic interaction between both implants, leading to imaging artifacts outside of the direct shadowed area. While MRI artifacts are expected with cochlear implants, the bilateral placement in this case appeared to exacerbate the effect, potentially due to device orientation or interaction. Had the patient not continued to recover and clearer imaging deemed vital, the internal magnet from the receiver-stimulator package of the cochlear implant could have been surgically removed. This can be performed under local anesthesia if required, and replacement of the magnet can be performed when the clinical situation allows.

To our knowledge, this is the first case reporting ischemic stroke following routine cochlear implantation in a patient with MELAS. Special considerations must be undertaken perioperatively for patients with MELAS. Perioperative body temperature control is critical, as hyperthermia can precipitate metabolic crises, while hypothermia may further impair mitochondrial function [12]. The choice of anesthetic is especially critical given that agents such as propofol can lead to the inhibition of mitochondrial enzymes [13]. A recent case report detailed the use of remimazolam and reversal with flumazenil for a patient undergoing cochlear implantation with an underlying mitochondrial disorder. In this case, remimazolam was used due to its short-acting profile and reduced mitochondrial toxicity, with flumazenil reversal ensuring rapid recovery [14]. Our patient, who was not yet diagnosed with a mitochondrial disorder at the time, underwent two procedures requiring propofol anesthesia (cochlear implantation and embolectomy). Propofol is generally contraindicated in MELAS due to its inhibitory effects on mitochondrial enzymes and may have contributed to her negative outcome. While case reports have detailed the use of propofol and remifentanil for patients with MELAS, this was limited to short procedures, approximately one hour in length, and may not be applicable to longer surgeries [12].

## Conclusions

Cochlear implantation remains a viable option for SNHL in MELAS, though surgical risks and anesthetic considerations require careful management. However, meticulous perioperative planning, including metabolic monitoring, cardiovascular stabilization, and anesthetic choice, is essential to minimize complications associated with MELAS.

This case highlights the importance of robust preoperative screening for mitochondrial disease in patients with unexplained metabolic abnormalities, particularly to mitigate anesthesia-associated risks. Additionally, given the increased stroke risk associated with MELAS-related vasculopathy, strict intraoperative blood pressure control is crucial to reduce the risk of potential cerebrovascular complications. We recommend that preoperative biochemical and genetic testing should be considered in high-risk patients to facilitate early diagnosis and to facilitate anesthetic and perioperative strategies that enhance patient safety.
